# Identifying Key Predictors of Neurodevelopmental Sequelae Absence at 12 Months in Infants with Congenital CMV: Insights from a Single-Center Retrospective Study

**DOI:** 10.3390/children13091199

**Published:** 2026-09-05

**Authors:** Alessia Spadavecchia, Carlotta Rubino, Agata Leone, Elena Maggiora, Chiara Peila, Francesco Cresi, Alessandra Coscia

**Affiliations:** Neonatal Care Unit, Department of Public Health and Pediatric Sciences, University of Turin, 10126 Torino, Italy; carlotta.rubino@unito.it (C.R.); agata.leone@unito.it (A.L.); elena.maggiora@unito.it (E.M.); chiara.peila@unito.it (C.P.); francesco.cresi@unito.it (F.C.); alessandra.coscia@unito.it (A.C.)

**Keywords:** cCMV, prognostic factors, neurodevelopment, SNHL

## Abstract

**Highlights:**

**What are the main findings?**

- Normal neutrophil count at birth is associated with the absence of neurodevelopmental sequalae at 12 months of age in children with congenital CMV infection.
- Normal audiological assessment at birth is associated with the absence of neurodevelopmental sequalae at 12 months of age in children with congenital CMV infection.

**What are the implications of the main findings?**

- The results of our study highlight new potential predictive factors for normal neurodevelopmental outcome in children with congenital CMV infection.
- It is necessary that newborns with congenital CMV infection undergo an extensive assessment at birth in order to properly evaluate all potential neurodevelopmental predictive factors and therefore improve clinical management.

**Abstract:**

Background: Congenital cytomegalovirus (cCMV) is the most frequent infective cause of morbidity and long-term sequelae in infancy. Despite great attention to this clinical condition, potential prognostic factors for neurodevelopmental sequelae in cCMV patients are poorly understood, therefore hampering optimization of clinical care and management of cCMV patients. Methods: A retrospective single-center cohort study was conducted on 66 infants with congenital CMV infection in order to identify among clinical presentation at birth potential predictors of the absence of neurodevelopmental sequelae at 12 months of age. A logistic regression model was built in order to identify a correlation between potential predictors and neurodevelopmental status at one year of age in the cohort in the study. Results: The results of our study showed a significant correlation between normal neutrophil count and normal audiological assessment at birth in a logistic regression model (*p* < 0.05) in patients with cCMV with the absence of neurodevelopmental sequelae at M12. Our findings also underlined that normal platelet count and normal cerebral imaging are connected with normal neurodevelopment at one year of age, though not in a statistically significant way. Conclusions: Our findings underscore the need for appropriate assessment at birth in order to improve clinical management of cCMV patients.

## 1. Introduction

Congenital cytomegalovirus (cCMV) is one of the most common and potentially health-impacting viral infections in infants [1]. A great deal of attention has been focused on CMV in recent years, which has allowed for a better understanding of the course of this disease and new clinical advances. Despite all the scientific advancements and new evidence, one of the areas where uncertainties remain is in the long-term clinical outcomes of affected children, particularly those who are asymptomatic or mildly symptomatic at birth [2].

The clinical manifestations of cCMV cover a broad spectrum of symptoms, from asymptomatic cases to patients with severe hearing and neurodevelopmental impairment [3]. The peculiarity, however, is that even in asymptomatic infants at birth, long-term sequelae may occur. Indeed, about 10% will develop sensorineural hearing loss (SNHL), whereas no differences from the healthy population have been reported in terms of neurodevelopment [4]. Recent studies have also highlighted the risk of developing vestibular sequelae and behavioral alterations [5,6].

The introduction of Valacyclovir for secondary prevention in pregnancy led to the modification of screening policies by introducing universal screening for CMV in pregnant women [7,8]. To date, however, newborn screening is not recommended because it raises numerous questions regarding its effectiveness, cost, and ethical implications, although it would be useful to ensure early identification of infected infants and enable monitoring for long-term sequelae and timely interventions [9].

Valganciclovir is the approved treatment for neonates with cCMV, but it is not offered to all affected infants. Instead, it is reserved for those who are most severely compromised by the virus, in whom long-term complications are more likely [8].

Therefore, understanding which infants are at risk of developing sequelae is crucial.

Prognostic factors influencing the general and neurodevelopmental outcome in infancy for patients with cCMV are still to be fully characterized and are currently the object of study. Several experimental factors linked to the viral behavior of CMV, differences in CMV genomic variability and interaction of CMV with the host immune system [10], metabolomic CMV impact [11] and exosomal miRNA expression [12] have been characterized so far, but studies on larger population samples are needed to confirm these findings.

Comprehensive knowledge about at-risk infants, even those mildly symptomatic, may allow us to offer the correct family counseling, identify which infants would benefit from early therapeutic interventions, and in the future may enable us to take advantage of universal newborn screening.

The aim of this study is to identify prognostic factors that may predict favorable long-term outcomes in infants with congenital CMV infection.

## 2. Materials and Methods

**Study Design and Population**: The presented monocentric retrospective cohort study enrolled patients with diagnosed cCMV infection at birth, followed in the Neonatal Intensive Care Unit of the University, Sant’Anna Hospital, Turin, from 2005 to 2023.

Inclusion criteria for the study comprised being diagnosed with congenital CMV infection at birth, the absence of maternal treatment with valaciclovir and completion of at least one year of follow-up.

Patients with incomplete follow-up or missing data were not included in the study.

Informed consent was obtained from the parents of the children recruited in this study.

Maternal infection was diagnosed via serological analysis and further characterized on the basis of serology results as primary infection (MPI) or non-primary infection (MNPI), wherever data were available. MNPI was defined in cases of identification of rise in specific IgG titer.

CMV infection in neonates was diagnosed by detecting CMV DNA in urine through PCR. This diagnostic test was performed in cases where there was maternal infection evidence or when neonates exhibited cCMV suggestive symptoms.

**Neonatal assessment and follow-up**

A routine laboratory analysis, including bone marrow, liver and kidney function evaluation, was performed at birth for every patient in the study.

In particular, the absolute neutrophil count was considered normal above 1000 cells/mm^3^, as stated in the recently published European Consensus about cCMV [8].

The patients underwent follow-up evaluations at 1, 3, 6 and 12 months of age, which comprised clinical evaluation, anthropometric measurements and neurodevelopment evaluation. In case of the need for antiviral therapy with valganciclovir during the first six months of life, the patients also received two additional follow-up evaluations at 18 and 24 months old. Griffith II/III was performed at M12 and M24 whenever possible. Cerebral palsy, cognitive impairment, speech and language delay, global motor delay, and hemiplegia were considered as neurodevelopmental sequelae and therefore indicative of poor neurological outcome.

Concerning instrumental assessments, all patients underwent both cerebral ultrasound and MRI in the first month of life, with potential repetition of the said exams on the basis of the anomalies detected and clinical examination.

All the patients also underwent audiological assessment comprising otoacoustic emission testing, auditory brainstem response and auditory steady state response during the clinical follow-up. Vestibular function assessment was not performed systematically and started to be performed routinely in 2023.

The cohort of patients in the study also underwent ophthalmological evaluation at M1 and M12 of age.

**Statistical analysis:** Categorical variables were expressed as the number of patients and percentage of patients. Numerical variables, where available, were described using mean and standard deviation. To evaluate the association between the absence of symptoms and the absence of neurodevelopmental sequelae at M12, a logistic regression model was built. The association between variables and the outcome was estimated by crude OR and adjusted OR, with a 95% confidence interval.

## 3. Results

**Figure 1:**
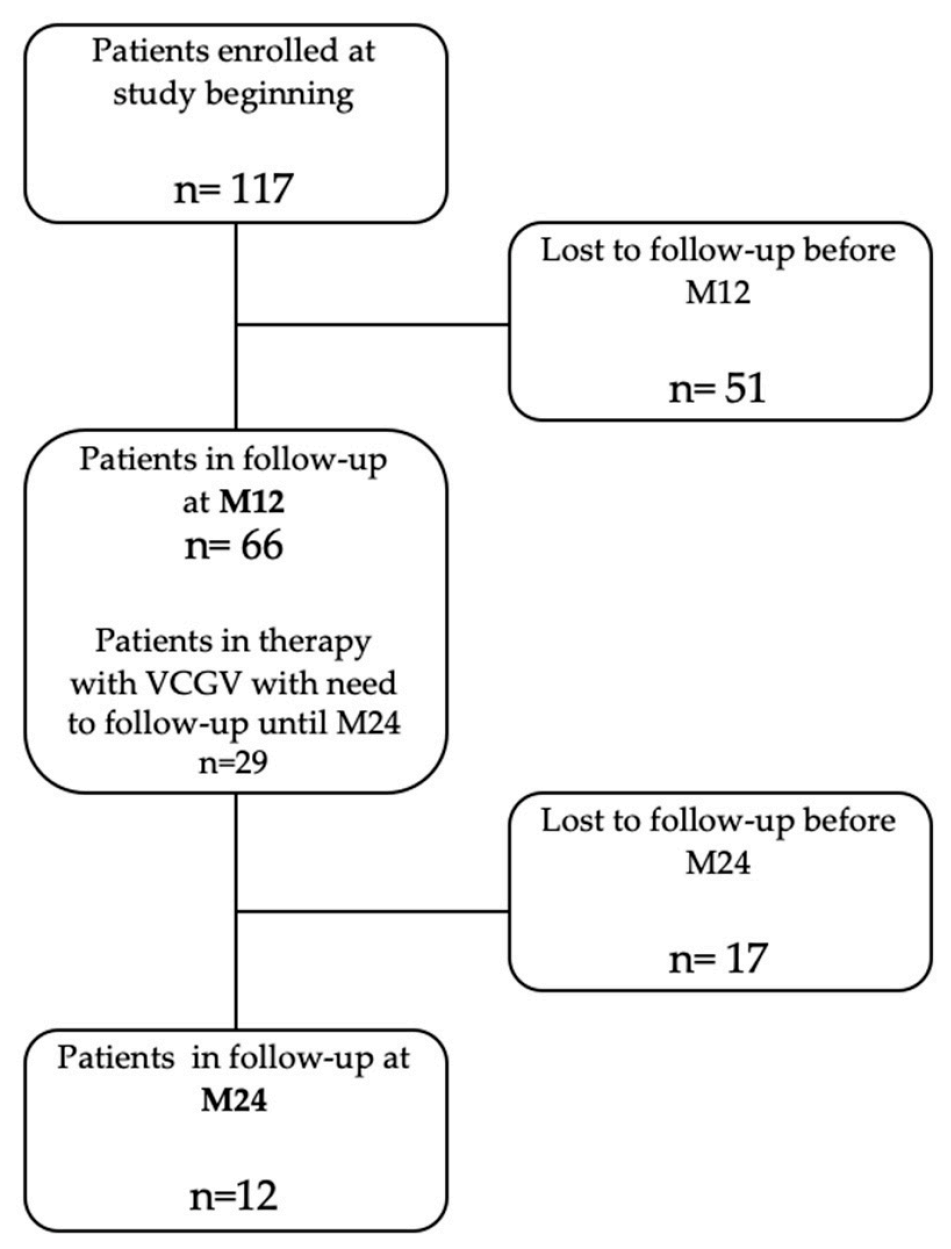
Descriptive flowchart of the population in the study through the different timepoints.

A total of 117 cCMV patients were enrolled in the study; 66 (56.4%) of the patients were followed up until M12, while 12 (10.2%) of them were followed up until M24 (as summarized in Figure 1). We therefore considered the 66 patients who completed the follow-up at M12 as the study cohort. A baseline descriptive analysis of the study population is summarized in Supplementary Materials Table S1.

Concerning maternal infection, cCMV was subsequent to a maternal primary infection (MPI) in 28 (42.4%) of the patients, while a maternal non-primary infection (MNPI) occurred in three (4.5%) of the patients. Data regarding the type of maternal infection were not available in 35 patients (53%).

In 89.2% (25/28 patients) of the cases of MPI and in 66.6% (2/3 patients) of the cases of MNPI, the patients did not display neurodevelopmental sequelae at M12. Maternal CMV infection occurred during the first trimester of gestation in 21.2% of the patients (14/66 patients) and during the second or third trimester in 25.7% of the cases (17/66 patients). In 53% of the newborns (35/66 patients) in the study, it was not possible to date maternal infection. In 71.4% of the cases of cCMV that occurred during I trimester of gestation (10/14 patients) and in 100% of the cases of cCMV that occurred during II or III trimester (17/17 patients), patients did not display neurodevelopmental sequelae at one year of life.

Concerning clinical presentation at birth, the cohort of patients in the study did not present any clinical symptoms (petechiae, hepatomegaly, splenomegaly, chorioretinitis, seizures, hypotonia) in 78.7% of the cases (52/66 patients). Among those patients, neurodevelopmental outcome at M12 was normal for age in 92.3% of the cases (48/52 patients). Patients with symptomatic presentation at birth accounted for 21.2% of the cases (14/66 patients), and 42.9% of the cases (6/14 patients) displayed sequelae at M12. The newborns in the study presented normal platelet count at birth in 68.1% of the cases (45/66 patients) and normal absolute neutrophil count (ANC) in 63.6% of the cases (42/66 patients): neurodevelopment at M12 was normal in 86.7% (39/45 patients) and 90.4% of the patients (38/42), respectively. Concerning instrumental exams, the patients presented normal cerebral ultrasound (US) in 48.5% of the cases (32/66 patients), with normal neurodevelopment at M12 in 100% of the newborns. Cerebral magnetic resonance imaging (MRI) was normal in 37.9% of patients evaluated (22/25 patients): in 88% of the cases, neurodevelopmental outcome was normal.

Concerning audiological assessment at birth, 35.0% of patients had normal audiological examinations (49/66), with 96% of them presenting normal neurodevelopment at M12 (47/49 patients). The description of the population in the study is shown in Table 1.

A logistic regression model was built in order to study the correlation between neurodevelopmental outcome and potential predictors among the clinical presentation at birth. The population considered for the model was composed of 66 patients who successfully completed follow-up at M12. The model revealed that normal ANC and normal audiological assessment at birth were significantly predictive for normal neurodevelopmental outcome at one year of life (*p* < 0.05). Despite being not statistically significant, it is important to note that normal platelet count and normal cerebral imaging are also associated with a normal neurodevelopment at M12, as shown by the crude and adjusted OR.

The results obtained in the elaboration of the logistic regression model are shown in Table 2.

A second logistic regression model was also built in order to estimate the correlation between audiological status and symptoms at birth and audiological status at M12. The model revealed that normal hearing at birth was significantly predictive of normal hearing at one year of life. Despite not being statistically significant, the absence of clinical symptoms at birth also relates to normal hearing at M12.

The results obtained in the elaboration of logistic regression model are shown in Table 3.

## 4. Discussion

CMV infection in neonates is a significant concern due to its potential long-term sequelae. Recent research efforts are increasingly focused on identifying prognostic factors at birth that can predict a more severe clinical manifestation of the infection and long-term sequelae.

Concerning maternal factors, scientific evidence has shown through the years the impact of trimester of infection on the clinical outcome of patients with cCMV, highlighting it as one of the most important prognostic factors so far. In particular, first-trimester infections correlate with severe clinical outcomes at birth and long-term sequelae in infancy [13]. Despite this, the total absence of outcomes in infections in the second and third trimesters is controversial. In fact, cases of SNHL are also reported in infections in trimesters other than the first [14]. Our study supports the finding that CMV infection during the first trimester is associated with poorer outcomes at 12 months, whereas infections occurring in the second and third trimesters do not result in sequelae at 12 months. This finding points out the importance of routine serological screening for CMV in pregnancy in order to identify all first-trimester infections and provide appropriate therapy during pregnancy.

Regarding neonatal factors as predictors of long-term outcomes that have been reported in the literature so far, neuroimaging has an important role. An encephalic alteration on prenatal imaging appears to be associated with an increased likelihood of cochleovestibular dysfunction later in life [15]. Alterations on cranial ultrasound scanning performed at birth often result in correlation with later neurological outcomes in affected infants [16]. Lastly, brain MRI results in being a valuable prediction tool, especially when specific scores are used [17]. Additionally, a normal brain MRI often predicts a favorable prognosis, underscoring the importance of this modality in the early evaluation of the newborn infant with cCMV [18].

With regard to clinical manifestations, certain signs at birth have been shown to be significant indicators of potential sequelae. For example, the presence of petechiae appears to be predictive of an increased risk of developing congenital hearing loss [11]. On the neurodevelopmental side, a particularly significant predictor is microcephaly at birth [19]. Still, a low viral load in the infant’s blood has been correlated with better clinical outcomes [20]. A recent French study developed a model based on three variables: normal hearing at birth, normal platelet count at birth, and normal cranial ultrasound at birth that proved accurate in predicting the absence of long-term sequelae [21].

Concerning clinical presentation at birth, our study shows a significant correlation between the absence of neurodevelopmental sequelae at 12 months and normal neutrophil count and normal hearing assessment at birth. A potential biological explanation could be linked to different virological behavior between CMV strains, with different abilities to involve different systems (including the inner ear and bone marrow). These differences in virological behavior have already been demonstrated in in vitro and animal models, but further studies are currently needed in order to confirm these hypotheses [10,22]. Additionally, a normal platelet count and normal brain MRI at birth also suggest better outcomes, but these correlations are not statistically significant. Regarding the audiological outcomes, our study also shows a significant correlation between normal hearing at birth and normal hearing at 12 months of life. Nonetheless, despite not being statistically significant, the absence of clinical symptoms at birth is also correlated with normal hearing at M12.

Our study strengthens the necessity, already expressed in the literature, of appropriate diagnosis and adequate assessment at birth, in order to identify patients at higher risk of sequelae and initiate appropriate interventions in order to achieve the best possible neurodevelopmental outcome.

The limits of our study are represented by the small sample size and study design. In the first case, because of the small number of events, the logistic regression model was restricted to four independent variables (normal platelet count at birth, normal ANC at birth, normal cerebral MRI, normal audiological assessment) in order to obtain reliable estimates. Also, for the same reason, the evaluation of the association between variables and neurodevelopmental sequelae was performed at M12 and not at M24. Concerning the study design, the retrospective design takes into account the occasional lack of some specific data of the patients in the study. Therefore, a prospective study is needed to expand the knowledge about predictors of the presence and absence of neurodevelopmental sequelae at M12 and M24 in cCMV patients.

Further evidence is needed to confirm or reject these hypotheses.

## 5. Conclusions

The results shown in the present study identify normal ANC and normal audiological examination at birth as potential key predictive factors for normal neurodevelopment of patients with cCMV, therefore providing important evidence for the correct management of these patients and improving the knowledge regarding positive prognostic factors in this specific clinical condition.

## Figures and Tables

**Table 1:** Descriptive analysis of the population in the study.

	Total	No Sequelae at M12	Presence of Sequelae at M12
**Type of infection**			
Primary Infection	28/66 (42.4%)	25/28 (89.2%)	3/28 (10.7%)
Non-primary Infection	3/66 (4.5%)	2/3 (66.6%)	1/3 (33.4%)
Data not available	35/66 (53%)		
**Trimester of infection**			
I trimester	14/66 (21.2%)	10/14 (71.4%)	4/14 (28.9%)
II-III trimester	17/66 (25.7%)	17/17 (100%)	0/17 (0%)
Data not available	35/66 (53%)		
**Neonatal assessment**			
Absence of clinical symptoms at birth	52/66 (78.7%)	48/52 (92.3%)	4/52 (7.7%)
Normal platelet count at birth	45/66 (68.1%)	39/45 (86,7%)	6/45 (13.3%)
Normal ANC	42/66 (63.6%)	38/42 (90.4%)	4/42 (9.6%)
Normal cerebral US	32/66 (48.5%)	32/32 (100%)	0/32 (0%)
Normal cerebral MRI	25/66 (37.9%)	22/25 (88%)	3/25 (12%)
Normal audiological examination	49/66 (74.2%)	47/49 (96%)	2/49 (4%)

**Table 2:** Logistic regression model assessing correlation between neonatal predictors and absence of neurodevelopmental sequelae at M12.

	Absence of Sequelae at M12	Crude OR (95% CI)	Adjusted OR (96% CI)	*p* Value
Normal platelet count Yes No	39/45 1/2	10.250 [0.553–196.963]	2.33 [0.629–5.280]	0.123
Normal ANC Yes No	38/42 4/7	7.125 [1.576–43.853]	1.96 [0.146–3.780]	0.034
Normal cerebral MRI Yes No	22/25 12/18	3.194 [0.949–15.703]	1.16 [−0.431–2.754]	0.153
Normal audiological examination Yes No	41/43 5/12	21.467 [4.175–110.376]	3.07 [1.43–4.70]	<0.001

**Table 3:** Logistic regression model assessing correlation between neonatal predictors and normal hearing at M12.

	Absence of SNHL at M12	Crude OR (95% CI)	Adjusted OR (95% CI)	*p* Value
Absence of clinical symptoms at birth Yes No	35/42 1/3	8.750 [0.704–108.787]	2.17 [−0351–4.689]	0.092
Normal audiological examination at birth Yes No	35/37 2/9	61.250 [7.342–510.943]	4.11 [1.99–6.24]	<0.001

## Data Availability

The original contributions presented in this study are included in the article/Supplementary Materials. Further inquiries can be directed to the corresponding author.

## Supplementary Materials

The following supporting information can be downloaded at: https://www.mdpi.com/article/10.3390/children13091199/s1. Table S1 Additional descriptive analysis of the population in study.
