# Supplementary material for: Identifying Key Predictors of Neurodevelopmental Sequelae Absence at 12 Months in Infants with Congenital CMV: Insights from a Single-Center Retrospective Study
Source: Children (Basel). 2026 Sep 5;13(9):1199. doi: 10.3390/children13091199 (PMC13605399; doi:10.3390/children13091199)
Supplement: Supplementary file 1 [file children-13-01199-s001.zip › children-4460603-supplementary.pdf]

Supplementary Materials

Table S1. Additional descriptive analysis of the population in study.

ADDITIONAL GENERAL DESCRIPTIVE ANALYSIS			
**Type of delivery**		**Necessity of reanimation at birth**	
Spontaneous delivery	23/66	Yes	6/66
Cesarean section	43/66	No	60/66
**Preterm birth**		**Mean birth weight (gr)**	2783.01 ± 690.38
Yes	8/66	**Mean length at birth (cm)**	43.63 ± 3.59
No	58/66	**Mean head circumference (cm)**	31.23 ± 6.75
